# Discrepancy of Beta-Hydroxybutyrate Measurements between a Blood Meter and GC-MS Methods in Healthy Humans

**DOI:** 10.3390/muscles2040025

**Published:** 2023-09-27

**Authors:** Angelia Maleah Holland-Winkler, Andrew R. Moore, Jenna K. Ansley, Noah A. Fritz, Ilya Bederman

**Affiliations:** 1 Department of Kinesiology, Augusta University, Augusta, GA 30909, USA; andmoore@augusta.edu (A.R.M.);; 2 Department of Genetics and Genome Sciences, School of Medicine, Case Western University, Cleveland, OH 44106, USA; irb2@case.edu

**Keywords:** β -hydroxybutyrate, blood meter, humans, ketone salts, ketosis

## Abstract

Ketone salt (KS) supplementation induces temporary nutritional ketosis to achieve potential exercise performance and health benefits. Racemic KS includes both D/L isomers of β-hydroxybutyrate, yet commercially available measurement devices (i.e., blood meters) only measure the D variant. The aim of this study was to investigate the efficacy of a blood meter to measure serum β-hydroxybutyrate in comparison with gas chromatography–mass spectrometry (GC-MS) before and 30 min after consuming a placebo or racemic KS. In this triple-blinded cross-over study, 16 healthy adults were administered either a placebo or KS drink, and the circulating β-hydroxybutyrate concentration was measured at baseline (PRE) and 30 min following consumption (POST) using a blood ketone meter and by GC-MS. Compared to the placebo, both GC-MS and the blood meter obtained significantly greater β-hydroxybutyrate levels from PRE to POST time-points after consuming KS. Additionally, GC-MS results showed significantly higher levels of β-hydroxybutyrate with both the placebo and KS at PRE and POST time-points, as compared to the blood meter. These results indicate that (1) even in the absence of KS, the blood meter yields significantly lower β-hydroxybutyrate values than GC-MS, and (2) the inability of the blood meter to measure L-β-hydroxybutyrate values POST KS warrants the further development of publicly available ketone measurement apparatuses.

## 1. Introduction 

Ketosis is a metabolic state facilitated by a variety of circumstances, including intermittent or prolonged fasting, starvation, prolonged exercise, uncontrolled type 1 diabetes mellitus, or a ketogenic diet consisting of little to no carbohydrates (<20–50 g/day) [1,2]. Under each of these conditions, circulating glucose available for ATP synthesis becomes sparse, insulin levels fall, and, as a result, there is a significant increase in the lipolysis of adipose tissue triglycerides to provide free fatty acids for β-oxidation and ATP synthesis. However, certain tissues cannot metabolize long-chain fatty acids, relying solely on glucose for their ATP production, and thus require another source of fuel (the primary examples being erythrocytes and neurons, respectively) [3,4,5]. Hepatic β-oxidation produces excess NADH + H^+^, which inhibits Krebs cycle activity as well as the pyruvate dehydrogenase enzyme complex, thus diverting pyruvate towards gluconeogenic oxaloacetate. As the Krebs cycle flux is inhibited, acetyl-CoA generated from β-oxidation accumulates and is converted into ketone bodies, a process otherwise known as ketogenesis [1,6]. The liver is the only organ to produce ketone bodies, namely acetoacetate (usable form) and beta-hydroxybutyrate (βHB) (transport form), although the liver does not use ketone bodies for energy since it is missing a key enzyme for ketone body utilization, β-ketoacyl-CoA transferase [7]. During glucose deprivation, ketone bodies become the primary source of fuel for the brain, as they are easily transported and diffusible across the blood–brain barrier via monocarboxylate transporters 1, 2, and 4 [8,9]. Acetone, a ketone body formed from the spontaneous decarboxylation of acetoacetate, has not been shown to contribute to the adaptations associated with ketosis, although it displays some anticonvulsant properties in rat seizure models [7,10]. 

Extrahepatic organs utilize ketone bodies for fuel, with the oxidation of βHB occurring in the mitochondrial matrix. Skeletal muscles with high mitochondrial content are a primary tissue for βHB utilization at rest [10]. Endurance training has been shown to increase the concentration of βHB in skeletal muscle and increase the mitochondrial enzymatic capacity, which further elevates βHB uptake and utilization during exercise [11,12]. The oxidation rate of βHB was higher in endurance-trained mice than in sedentary mice. βHB may improve athletic performance and myocardial ATP generation by modifying mitochondrial function [13]. For example, βHB has been shown to improve muscle cell function by increasing mitochondrial respiration without reducing ATP production [14].

Endogenous βHB is the D isomer (D-βHB), while exogenous ketone supplementation with synthetically produced KS often contains a racemic mixture of both the D and L isomers, the latter of which was shown to be partially converted into D-βHB in rat livers [15]. Beyond this, little research has investigated the physiological utilization of L-βHB in any animal model, other than the observation reported by Webber and Edmond in 1977, showing that, in rats, while D-βHB is the favored substrate for oxidation (i.e., ketolysis), L-βHB is favored for the synthesis of sterols and fatty acids in periods of neonatal development [16]. Additionally, Stubbs et al., 2017 found that, in healthy adults, L-βHB consumed as a racemic KS supplement was metabolized slower than its isomeric counterpart, and that which failed to convert to D-βHB was excreted in urine [17]. The metabolic fate of each isomer is important, as exogenous ketone supplementation with KS has been shown to elevate serum ketone levels and lower blood glucose [17,18], and the ketone measurement apparatus most available to the public—a blood meter—only measures the concentration of the D-βHB isomer. 

The efficacy of any ketone measurement apparatus publicly available is ultimately determined by its output compared to that of a gas chromatographer–mass spectrometer (GC-MS) system, which simultaneously measures both acetoacetate and βHB in a single analysis. GC-MS detection of molecules is based on the unique behavior of ionized molecules and their pattern-forming abilities. Briefly, the gas chromatography part of GC-MS can separate molecules in a complex sample matrix (i.e., blood) based on their boiling point, volatility, and polarity. Next, the mass spectrometry part of GC-MS ionizes the molecules to form a unique fragmentation pattern, which is used to identify molecules with 1Da precision. GC-MS has long been considered the most valid and accurate technique with regard to ketone metrics [19], and the importance of establishing accurate measurements of circulating ketone bodies lies in its potential to help to better discern between states of nutritional ketosis (read as 0.5–3 mM when using a blood meter) and pathological ketosis, otherwise known as ketoacidosis, a state that exceeds nutritional ketosis by 5–10-fold greater values of βHB [20,21]. Ketoacidosis is induced either by uncontrolled type 1 diabetes mellitus with a complete lack of insulin signaling or extreme alcohol consumption paired with severe caloric restriction [20,21]. Blood meters, such as the Abbott Precision Xtra, are commonly used as a point-of-care ketone body assessment tool in clinical situations for the diagnosis of ketoacidosis. They are deemed appropriate for this purpose because, although not as accurate as reference methods, they provide nearly instantaneous results at a lower cost, which allows clinicians to make critical healthcare decisions based on more frequent ketone readings [22]. 

However, no studies to date have focused on investigating the validity of commonly used blood meters to measure βHB in healthy humans. Additionally, no studies have addressed the potential discrepancy in βHB measurement following the consumption of racemic KS, which is increasingly used by athletes and the general public to induce a state of nutritional ketosis for a variety of purposes. Therefore, the primary aim of this study was to investigate the efficacy of a blood meter to measure serum βHB in comparison with GC-MS following the consumption of a racemic KS supplement or a placebo in healthy humans. The secondary aim was to investigate the efficacy of a blood meter to measure endogenous serum βHB in comparison with GC-MS in people in a fasted and resting state prior to any supplementation. We hypothesized that the total βHB levels may differ between the devices following the exogenous KS ingestion, and that GC-MS readings would be greater than blood meter readings of βHB under the same conditions.

## 2. Results

A three-way repeated-measures ANOVA was conducted to determine the effects of drink, device, and time on blood βHB levels. There was a statistically significant three-way interaction between device, time, and drink, F(1, 13) = 12.12, *p* = 0.004. 

At the PRE time-point, βHB was significantly higher when measured with GC-MS than with the blood meter in both the KS (0.752 ± 0.290 vs. 0.229 ± 0.184, g = 2.15) and placebo (1.056 ± 0.861 vs. 0.218 ± 0.209, g = 1.34) conditions (*p* < 0.001 in all cases). Similar results were found for the POST time-point in both the KS (3.109 ± 2.120 vs. 0.468 ± 0.227, g = 1.75) and placebo (0.806 ± 0.331 vs. 0.179 ± 0.124, g = 2.51) conditions (*p* < 0.001 in all cases).

**Figure 1:**
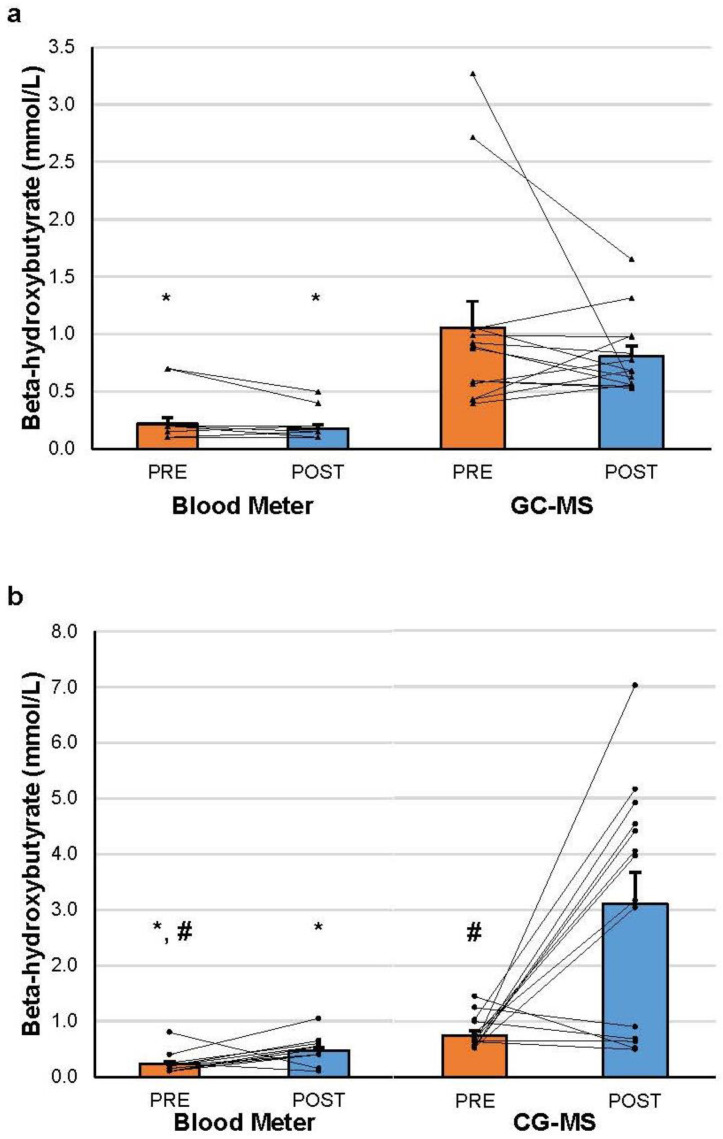
(**a**) βHB values before and after placebo supplementation; (**b**) βHB values before and after ketone salt supplementation. For each graph, bars display the mean value; whiskers display the standard error of the estimate; and markers represent individual values for PRE and POST conditions, connected by a line to display individual changes in beta-hydroxybutyrate. Abbreviation: GC-MS, gas chromatography–mass spectrometry. * = significantly different from gas chromatography–mass spectrometry value at the same time-point and in the same supplement treatment. # = significantly different from POST value in the same supplement condition and with the same device.

After KS administration, βHB significantly increased from PRE to POST for both the blood meter (*p* = 0.013, g = 1.15) and GC-MS (*p* = 0.002, g = 1.55). βHB was significantly higher in the GC-MS than the blood meter condition at the POST time-point (*p* < 0.001, g = 1.75). Individual and group changes in βHB are represented graphically in Figure 1a,b. Complete descriptive data and statistical results can be found at an online repository using the following link, which was last updated on 18 August 2023: https://osf.io/fzxqn/.

**Figure 2:**
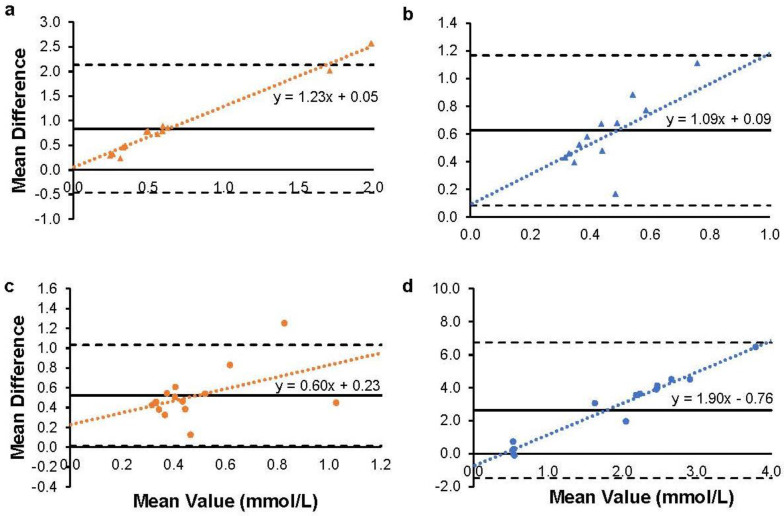
Bland–Altman plots showing agreement in βHB measurement between blood meter and GC-MS devices for placebo ((**a**)-PRE; (**b**)-POST) and ketone ((**c**)-PRE; (**d**)-POST) conditions. Each subject is represented by a marker plotted on the graph, with the x-value as the average βHB reading of the two devices and the y-value as the difference between the two devices (GC-MS-blood meter) for the respective reading. A y-value closer to 0 indicates a higher level of agreement. The level of agreement is assessed throughout the range of average βHB levels analyzed. The solid line represents the bias (average difference in measurement). The two dashed lines represent the limits of agreement (bias ± 2SD of bias). The dotted line is the line of best fit and is described by the linear equation present.

There was a substantial level of bias between the blood meter and GC-MS methods of measurement, as displayed in Figure 2. The bias ranged from 0.52 to 2.64 mmol/L depending on the condition. The level of variability in the measurement between methods (upper and lower limits of agreement) was large relative to the level of measurement of βHB. The trendlines for each plot show an increasing level of disagreement between devices (i.e., more error) as the level of βHB increases.

**Figure 3:**
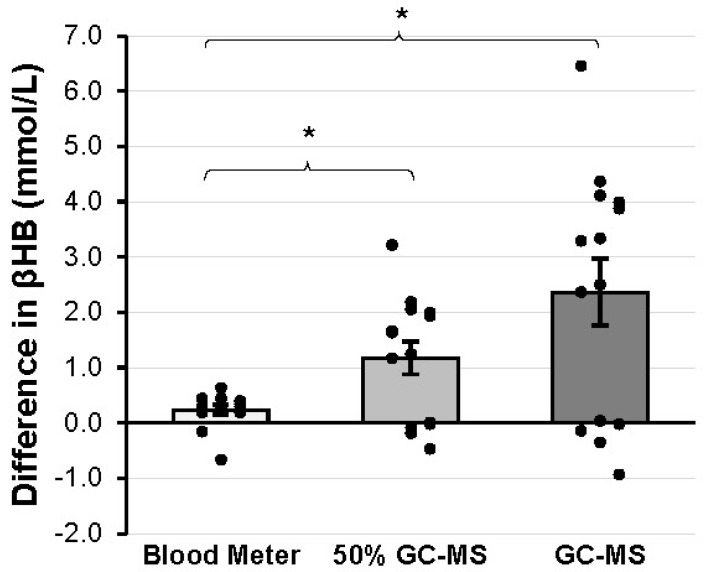
Differences in gain scores between devices. The change in βHB following ketone salt ingestion is compared between blood meter and GC-MS and between blood meter and 50% GC-MS, which is 50% of the gain score assessed with GC-MS for each subject. This adjusted 50% GC-MS value is used to account for the fact that only a possible 50% of the ketone salt supplement could be measured by the blood meter. * = significant difference between the pairs of gain scores indicated by brackets. Abbreviation: GC-MS, gas chromatography-mass spectrometry; 50% GC-MS, 50% of the βHB value measured by gas chromatography-mass spectrometry.

After the formal analysis of the results, a post hoc gain score analysis (comparing the changes in βHB value between blood meter and GC-MS from PRE to POST) was deemed appropriate to further investigate the nature of the differences in exogenous βHB measurement between devices. A significantly greater increase in βHB was detected when GC-MS (2.36 ± 2.25 mmol/L) was used compared to when the blood meter (0.24 ± 0.31 mmol/L) was used (*p* = 0.003, g = 0.92). An additional gain score comparison was made between the blood meter and 50% of the value attained from the GC-MS (50% GC-MS) to account for the fact that 50% of the KS supplement was of the L isomer, which cannot be measured by the blood meter. Thus, computing the gain score adjusted for the initial endogenous βHB and halving the GC-MS gain value adjusted for the L isomer that was included in GC-MS but not blood meter readings. βHB was greater in 50% GC-MS (1.18 ± 1.12 mmol/L) compared to the blood meter (*p* = 0.006, g = 0.831). These gain scores are represented graphically in Figure 3.

## 3. Discussion

The aim of this study was to compare the accuracy of a blood meter to measure serum βHB and GC-MS before and 30 min after having consumed either a mixed racemic KS supplement or a calorie- and flavor-matched maltodextrin placebo in fasted subjects. βHB levels that were measured by GC-MS were significantly and substantially higher than those measured by the blood meter in both the placebo and KS groups at both time-points. Both types of analyses detected a significant increase in βHB from PRE to POST in the KS group, but the magnitude of the increase in βHB was significantly greater with GC-MS. These findings support our initial hypothesis that these measurement methods would yield different results and that GC-MS would yield higher values of βHB. 

Compared to GC-MS, blood meters are cheaper and more widely available when measuring βHB. However, their ability to accurately measure βHB from exogenous sources (specifically KS) is in question. Previous studies have reported similar accuracy between these two devices for βHB values < 3.0 mmol/L [17,23]. However, these measurements were obtained in “patients with a routine laboratory request for blood ketone analysis” under unknown nutritional and clinical conditions. Additionally, only the accuracy of measuring D-βHB was assessed between the devices and the ability to detect exogenous βHB was not addressed. Other studies that compared βHB measurements using blood meters and GC-MS were performed in dairy cattle [24,25,26] and again did not consider the ability to measure exogenous ketones. This study is the first, to our knowledge, to examine βHB measurement discrepancies between a commonly used blood meter and GC-MS following racemic KS ingestion. 

The largest discrepancy between the two measurement devices was following the ingestion of KS. Blood meters can detect the presence of exogenous D-βHB in a dose-dependent manner [27], although the accuracy of these readings has not been validated against GC-MS in humans. A gain score analysis was used to determine the change in total βHB from resting fasted levels (endogenous βHB only) to 30 min post-consumption of the KS supplement. Thus, the resulting gain score was a measurement of the exogenous βHB concentration in blood. A significant and substantially larger increase in βHB was measured by GC-MS as compared to the blood meter. This discrepancy in βHB concentration between the devices was likely due to the inability of the blood meter to measure both the D and L isomers of βHB in the blood. The isomer D-βHB acts as an immediate fuel source, while the L-βHB may serve as a “preserved” ketolytic substrate that is converted to D-βHB at a slower rate [17]. Aside from being metabolic fuel, L-βHB may play other roles in the body, such as protection against oxidative damage during a hypoglycemic challenge [28] and appetite regulation as a means to induce weight loss when taken in the form of a ketone diester with D-βHB and acetoacetate [29]. Examining the effects of βHB on these processes using a blood meter that discounts the L-βHB isomer found in mixed racemic KS or ketone diesters may diminish the validity of experimental work. 

A secondary gain score analysis determined that when the gain in total βHB detected by GC-MS was halved (to account for the fact that half of this βHB increase was undetectable by the blood meter), the resulting increase in exogenous βHB was still significantly and substantially greater than that detected by the blood meter. The GC-MS sample analysis method used prevented the precise measurement of D-βHB, yet this estimation provides some evidence that the difference in βHB assessment between devices was not solely due to the inclusion of L-βHB. 

Differences between measurement devices were present even when no exogenous ketones were consumed—that is, even when the issue of L-βHB detection was not a concern. The differences between the blood meter and GC-MS in both PRE conditions suggests that there is a measurement discrepancy between devices even when only D-βHB is circulating and in the absence of any ingested substance, extreme fast, or clinical condition. The large differences between devices and the increasing error with higher βHB values displayed in Figure 2 emphasize these measurement discrepancies between the devices. This is relevant to those who routinely test their ketone levels in the morning (e.g., someone in the initial stages of a ketogenic diet) to determine when they have become keto-adapted. A commonly used cutoff point marking the onset of nutritional ketosis is a βHB value > 0.5 mM [20]. After a 10-h fast, the average βHB value for subjects in this study was above this cutoff as measured by GC-MS, but below the cutoff when measured with a blood meter. A closer examination of the data showed that in the PRE conditions, the result was a false negative for ketosis (a blood meter reading below 0.5 mmol/L with a GC-MS reading above 0.5 mmol/L) in 23 of 28 instances. The slightly lower sensitivity of the blood meter to detect endogenous βHB may lead to more false negative results for those interested in detecting nutritional ketosis and should be taken under consideration when using blood meters for this purpose.

The difference in βHB between the blood meter and GC-MS was present following placebo ingestion as well, showing that the discrepancy in βHB assessment between devices persisted after the fast was broken with maltodextrin. The ketone body concentration in the blood typically decreases upon carbohydrate ingestion, due to a decrease in the hepatic production of βHB [30]. This effect was observed when GC-MS was used, but not when the blood meter was used to assess βHB. People who test their βHB throughout the day to maintain a state of ketosis would benefit from recognizing this difference between devices. For example, βHB levels lower than expected during the day may prompt one to consume a greater volume of KS than is necessary or to restrict one’s carbohydrate intake to even more stringent levels than required. 

Supplementation with a racemic KS mixture may provide additional ketone bodies for use in the muscles and other tissues than previously understood, as all prior assessments with a blood meter have discounted the L-βHB isomer. Most of the ketone supplements available to the public exist in the form of KS, which includes both βHB isomers. Ketone esters and pure (100%) D-βHB KS are also in development, and research has already been published that illustrates their ketogenic efficacy [17,18,27,31,32]. Specifically, it is known that these supplements increase D-βHB concentrations faster and to higher levels than mixed racemic KS, and that this form of βHB is rapidly utilized for metabolic use. However, the price and availability make ketone esters and pure D-βHB unfeasible for many people, who may turn to mixed racemic KS instead. For this reason, understanding how to best assess and interpret the βHB readings from blood meters is important to those interested. At the very least, consumers of KS should be aware of the proportion of L-βHB in the product that they use and understand that this isoform yields different benefits from D-βHB. Manufacturers and consumers will be better armed to correctly interpret βHB readings from blood meters with such information.

βHB can be viewed as short-chain water-soluble fatty acids providing two Acetyl-CoA molecules per βHB molecule. This makes βHB particularly advantageous for skeletal muscle during exercise, when ATP requirements are high and there is a demand for quickly accessible energy sources. Since βHB is water-soluble and its entry into mitochondria is not controlled by the CPT-1 system, it becomes an important source of energy during exercise, thus sparing glycogen and potentially improving performance. During endurance exercise, βHB was shown to be released from the liver to supply working muscles [11]. Ketone metabolism adaptations occur in the skeletal muscle after regular endurance exercise training; specifically, skeletal muscle increases its capacity to utilize βHB for fuel, which leaves less βHB in the bloodstream post-exercise [12,14]. Sedentary individuals have substantially higher levels of circulating βHB after strenuous prolonged exercise than trained [12]. Trained individuals may find it difficult to maintain a βHB blood level associated with ketosis (0.5–3 mM) due to their enhanced capacity to utilize the βHB for fuel [13,20,21]. Ketone supplements are often used to increase the circulating supply of βHB to assist in the maintenance of ketosis. Blood meters are used to determine if ketosis is achieved; however, our results demonstrate that βHB levels may be higher than the blood meter reports, regardless of supplementation. Further explorations should identify the relationship between results from the blood meter and GC-MS at various blood βHB levels and synchronize levels associated with ketosis for each measurement method. 

One limitation of this study is that GC-MS only assessed total systemic βHB, and therefore was unable to provide specific values for both D-βHB and L-βHB. Future work will address this using special LC-MS (liquid chromatography–mass spectrometry) approaches to determine both D-βHB and L-βHB isomers. It may be beneficial to compare D-βHB readings, specifically, between devices to verify the accuracy of blood meters in recording βHB, which is used for metabolic purposes. Although it was not addressed in this study, the time course response of βHB is an area of interest as well. Persons taking KS would benefit from knowing the length of time that they would remain in a state of ketosis following supplement consumption. Future studies should investigate the serum levels and metabolic utilization of βHB over a prolonged period (1 h, 4 h, 12 h, etc.) following KS ingestion, given that L-βHB is metabolized more slowly and could yield prolonged benefits aside from metabolic fuel. Understanding that L-βHB is not reflected in blood meter readings is important, but the relevance of L-βHB to health and performance outcomes must be more broadly reported on to fully appreciate the applicability of the findings from this study. Therefore, more investigations into the signaling and metabolic roles of this isomer of βHB are needed. In addition, future explorations should compare blood meter and GC-MS measures of circulating βHB after consuming a ketone ester supplement. A final limitation of this study to consider is the sample size of 14 participants. The achieved statistical power was not a concern (Power > 0.81 for all ANOVA results), but the relatively low sample size limited the potential variability of the participants. A larger and more diverse sample for future studies may yield results that are more applicable to the general population.

## 4. Materials and Methods

### 4.1. Experimental Design 

A randomized, triple-blinded, placebo-controlled, cross-over design was used to compare blood meter and GC-MS measures of circulating βHB levels after consuming exogenous racemic KS. Participants visited the laboratory twice and consumed one of two supplements at random: placebo or racemic KS. Both supplements were calorie- and flavor-matched powders to be mixed with water. KS consisted of 7 g of racemic sodium D, L-βHB (50% D-βHB and 50% L-βHB), and a flavoring mixture, and the placebo consisted of maltodextrin, sodium, and flavoring. The proportion of each βHB isomer in the serving of KS was not known and could not be confirmed by the manufacturer. The supplements were coded as ‘A’ or ‘B’; the investigators and participants were blinded until the completion of the study. 

Prior to subject recruitment, subject numbers 1–16 were randomly assigned the order in which the placebo and KS would be provided at each of the two visits. The subject numbers were then assigned to participants in chronological order according to the signature date on the informed consent form. Participants visited the laboratory for a familiarization visit and then twice for data collection, with a one-week washout period between visits. The familiarization visit included signing an informed consent form, completing a health history questionnaire, and taking a pregnancy test if female. This study was approved by Augusta University’s Institutional Review Board, and all procedures performed followed institutional guidelines. 

### 4.2. Participants 

There were sixteen participants that completed the study, aged 18–35, recruited primarily from the university. The exclusionary criteria included (1) taking medications that affect blood pressure, insulin, or renal function; (2) metabolic syndrome factors such as type 2 diabetes; (3) pregnant; and (4) indicated on the health history questionnaire that there could be a pre-existing health condition.

Data loss for two of the participants resulted in their removal from the statistical analysis. Therefore, results for only 14 subjects are reported. The characteristics of the 14 participants with complete data are provided in Table 1.

### 4.3. Protocol 

Participants refrained from exercise, caffeine, and nicotine for 12 h and fasted for 10 h prior to initial data collection. Females took a pregnancy test before each data collection session. Height and weight were recorded at each visit. Baseline blood βHB was first measured with the blood meter (Precision Xtra™, Abbott Laboratories, Chicago, IL, USA). Specifically, the participant’s finger was cleaned with an alcohol wipe, pricked with a lancet, the first blood drop was wiped away, and the following blood drops were used for two blood meter measurements. Immediately following the blood meter measurements, the same procedures were followed for a second finger prick to collect a blood sample of 250 µL in a tube containing 0.2 mM lithium borodeuteride (685917, Millipore Sigma, St. Louis, MO, USA) to convert acetoacetate into [2-^2^H]βHB, and then immediately frozen on dry ice. Samples were stored at −80 °C until GC-MS analysis [33]. After baseline measurements, participants consumed their assigned supplement away from the testing site and washed their hands when done. POST-supplement measurements were taken 30 min after consuming the supplement. During the 30-min waiting period, participants were asked not to engage in physical activity. POST-supplement measurements were identical to PRE-supplement. The participants visited the laboratory again after a one-week washout period and the same procedures were followed with the other supplement. 

### 4.4. Sample Processing and GC-MS Conditions 

Samples were stored at −80 °C until analysis. Samples were thawed on ice and acidified by the addition of 1 N HCl. Internal standard ([2,4-^13^C_2_]βHB) was added, mixed, and extracted twice by the addition of 1 mL of an acetonitrile/isopropanol mix (1:1, vol/vol). Organic extracts were evaporated to dryness and βHB was converted to its trimethylsilyl derivative by reacting with 80 µL of bis(trimethylsilyl) trifluoroacetamide + 10% trimethylchlorosilane (Regis, Morton Grove, IL, USA) for 30 min at 75 °C. The di-trimethylsilyl derivative of βHB was analyzed using an Agilent 5973 mass spectrometer, linked to a 6890 gas chromatograph equipped with an autosampler, operated in electron impact ionization (EI) mode. The following ions were used in the analysis: βHB (*m*/*z* 233); M+1 ion (*m*/*z* 234), corresponding to [2-^2^H]βHB, representing the acetoacetate amount present in the sample after appropriate background subtraction; and M+2 ion (*m*/*z* 235) of the internal standard to calculate acetoacetate and βHB sample concentrations [34].

### 4.5. Data Analysis

All statistical analyses were performed using SPSS version 25. A predetermined alpha level of 0.05 was used for all analyses. The combined effects of drink (placebo or KS), device (blood meter or GC-MS), and time (PRE and POST) on the blood βHB level were analyzed using a 2 × 2 × 2 repeated-measures ANOVA. Post hoc tests with Bonferroni adjustment were performed as necessary to test for group differences following any significant interaction effects and main effects.

Outliers were operationally defined as data points with a standardized value > 3.0 from the group mean. Statistical analyses were completed with and without any outliers, to verify that the same statistical results were obtained in both cases. One outlier was identified for the βHB data, but its inclusion in the analysis did not change the outcome of any of the analyses. For this reason, the βHB results reported include this data point.

βHB values were assessed for normal distribution using the Shapiro–Wilk test of normality. The assumption of normality within each group was violated (*p* < 0.05), apart from one in the βHB analysis (blood meter, POST, KS). No data transformation or adjustments were made to account for these violations since the ANOVA is robust to non-normal data [35]. The Greenhouse–Geisser adjustment was applied to any data that violated the assumption of homogeneity of variance according to Levene’s test of equality of variances (*p* < 0.05). Data are reported as mean ± standard deviation unless otherwise stated.

The magnitude of the observed differences was assessed for each group by Hedges’ g (*g*). As proposed by Cohen [36], the magnitude of the effect (ES) was considered small (0.2 < ES ≤ 0.5), moderate (0.5 < ES ≤ 0.8), or large (ES > 0.8).

Bland–Altman plots were constructed for blood βHB measurements taken using the blood meter and GC-MS to assess agreement between devices. At each time-point (PRE and POST) and in each condition (placebo and KS), the readings for each device were used to calculate the bias (average difference between measurement devices) and upper and lower limits of agreement (bias ± 2SD of bias) between devices in measuring βHB across the range of observed values. The bias and limits of agreement were then used to construct the Bland–Altman plots to visually assess the agreement between devices at each time-point in each condition [37].

## 5. Conclusions

While blood meters are the most commonly used apparatus to assess systemic ketone bodies, their inability to detect L-βHB following the administration of mixed racemic KS means that they may underestimate the concentration of circulating βHB that is available for oxidation and tissue signaling. This is an important finding, given the prevalence of KS supplements in facilitating ketosis to improve exercise performance and other health-related outcomes. The use of blood meters to measure βHB in the absence of KS supplementation should also be approached with some caution, as they appear to be less sensitive than GC-MS, which is the most valid and precise method for βHB assessment. It may be more difficult to diagnose nutritional ketosis with a blood meter for this reason.

## Figures and Tables

**Table 1:** Summary of participant characteristics.

	Men (*n* = 7)		Women (*n* = 7)		Total (*N* = 14)	
	M	SD	M	SD	M	SD
Age (years)	22.57	(5.68)	20.43	(1.40)	21.5	(4.17)
Height (cm)	186.50	(11.58)	165.05	(10.93)	175.77	(15.52)
Weight (kg)	83.67	(13.71)	72.90	(22.44)	78.28	(18.72)
BMI (kg/m^2^)	24.43	(5.93)	26.53	(6.78)	25.48	(6.21)

## Data Availability

Complete descriptive data and statistical results can be found at an online repository using the following link, which was last updated on 18 August 2023: https://osf.io/fzxqn/.
